# Development of a pharmacoeconomic registry: an example using hormonal contraceptives

**DOI:** 10.1186/s13561-021-00309-z

**Published:** 2021-03-20

**Authors:** Annesha White, Meenakshi Srinivasan, La Marcus Wingate, Samuel Peasah, Marc Fleming

**Affiliations:** 1 University of North Texas System College of Pharmacy, University of North Texas Health Science Center, Fort Worth, TX 76107 USA; 2 Department of Pharmacotherapy, UNT System College of Pharmacy, 3500 Camp Bowie Blvd, IREB 211, Fort Worth, TX 76107 USA; 3 College of Pharmacy, Howard University, Washington, DC, 20059 USA; 4 Mercer University College of Pharmacy, Atlanta, GA 30341 USA

**Keywords:** Pharmacoeconomic registry, Hormonal contraceptives

## Abstract

**Background:**

Disease-specific registries, documenting costs and probabilities from pharmacoeconomic studies along with health state utility values from quality-of-life studies could serve as a resource to guide researchers in evaluating the published literature and in the conduct of future economic evaluations for their own research. Registries cataloging economic evaluations currently exist, however they are restricted by the type of economic evaluations they include. There is a need for intervention-specific registries, that document all types of complete and partial economic evaluations and auxiliary information such as quality of life studies. The objective of this study is to describe the development of a pharmacoeconomic registry and provide best practices using an example of hormonal contraceptives.

**Methods:**

An expert panel consisting of researchers with expertise in pharmacoeconomics and outcomes research was convened and the clinical focus of the registry was finalized after extensive discussion. A list of key continuous, categorical and descriptive variables was developed to capture all relevant data with each variable defined in a data dictionary. A web-based data collection tool was designed to capture and store the resulting metadata. A keyword based search strategy was developed to retrieve the published sources of literature. Finally, articles were screened for relevancy and data was extracted to populate the registry. Expert opinions were taken from the panel at each stage to arrive at consensus and ensure validity of the registry.

**Results:**

The registry focused on economic evaluation literature of hormonal contraceptives used for contraception. The registry consisted of 65 articles comprising of 22 cost-effectiveness analyses, 9 cost-utility analyses, 7 cost-benefit analyses, 1 cost-minimization, 14 cost analyses, 10 cost of illness studies and 2 quality of life studies. The best practices followed in the development of the registry were summarized as recommendations. The completed registry, data dictionary and associated data files can be accessed in the supplementary information files.

**Conclusion:**

This registry is a comprehensive database of economic evaluations, including costs, clinical probabilities and health-state utility estimates. The collated data captured from published information in this registry can be used to identify trends in the literature, conduct systematic reviews and meta-analysis and develop novel pharmacoeconomic models.

**Supplementary Information:**

The online version contains supplementary material available at 10.1186/s13561-021-00309-z.

## Background

In the field of health outcomes research, disease registries have been useful to collect clinical data, track patients over time and provide information for determining disease progression, safety and effectiveness. A registry is defined as “an organized system that uses observational study methods to collect uniform data (clinical and other) to evaluate specified outcomes for a population defined by a particular disease, condition or exposure, and that serves a predetermined scientific, clinical or policy purpose(s)” [1]. While the importance of clinically focused patient registries is well-defined, supplementing these registries with economic and quality of life (QOL) data provide an opportunity to assess cost-effectiveness of various interventions [2]. In contrast to patient registries, clinical trial outcome databases capture summary-level information from published randomized control trials (RCTs) and observational studies which can be used to perform network and model-based meta-analyses of safety and efficacy parameters [3]. The growth of the pharmacoeconomic literature over the past few decades has prompted several organizations to create databases or registries to compile key variables in published economic evaluations. Examples of current economic databases include the National Health Service (NHS) Economic Evaluations Database [4], Tufts CEA registry [5, 6], Tufts Global Health DALY (Disability-Adjusted Life Year) Registry [7], Pediatric Economic Database Evaluation Project [8], European Network of Health Economic Evaluation Databases (EURONHEED) [9] and the WHO Cost-Effectiveness and Strategic Planning (WHO-CHOICE) [10] databases. These registries are broad in their scope and represent economic evaluations conducted on a variety of healthcare, pharmaceutical and device interventions. Some of the aforementioned databases include different types of economic evaluations [4], while others are specific to cost-utility analysis [5] or cost per DALY studies [7]. However, there are only a few examples of comprehensive condition-specific health economic model registries, such as the Mount Hood Diabetes Challenge Network [11, 12]. Therefore, we sought to supplement the clinical literature on hormonal contraceptives (HC) with literature on all published complete and partial economic evaluations. Hormonal contraceptives were selected as a focus for a pharmacoeconomic registry for several reasons.

Globally, around 99 million pregnancies were unintended in 2010–2014 [13]. In the United States, nearly 45% of all pregnancies were unintended in 2011 [14]. Unintended pregnancies represent a major economic burden to the US public insurance programs, estimated to be $21 billion in 2010 [15]. In developing countries, women having an unmet need of contraceptives contribute to 84% of all unintended pregnancies, whereas the remaining occur among contraception users [16]. The reasons for contraception failure depend upon the inherent efficacy of the method, non-adherence, imperfect use, frequency of intercourse and age of the woman [17]. However, consistent use of contraceptives, still results in around 5% of unintended pregnancies in the USA [18]. Around 151 million women of reproductive age use hormonal contraceptive pills worldwide [19]. Recognizing the large public health implications, the FDA, the pharmaceutical industry and academia have focused efforts towards understanding the role of drug-drug interactions (DDIs) on HC failure [20]. Challenged by a lack of data on the clinical effect of DDIs associated with HCs, efforts are underway to study the impact of DDIs on HC failure from a multi-disciplinary perspective, including pharmacometrics, pharmacoepidemiology and pharmacoeconomics [21]. In order to evaluate the financial implications of unintended pregnancies resulting from drug-drug interactions with HCs, information was collated from all complete and partial economic evaluations on HCs. The present paper describes how the pharmacoeconomic clinical registry was developed and implemented, detailing its strengths and limitations with the aim of facilitating the creation of other condition specific pharmacoeconomic clinical registries. The registry structure and content are also presented along with best practice recommendations.

## Methods

This study was registered with the International Prospective Register of Systematic Reviews (PROSPERO CRD 42019118036) in January 2019. An expert panel consisting of researchers with expertise in pharmacoeconomics and outcomes research was convened. Based on the specific clinical question of pharmacoeconomics of HCs, the general approach to develop the registry was outlined.

### Environmental scan and study selection

A keyword-based search strategy was developed to identify relevant articles from PubMed and EMBASE. The search strategy was limited to consider women of reproductive age using hormonal contraceptives for the purpose of contraception, with the comparator being any other method of contraception or non-use of contraception. Studies assessing the impact of family planning interventions where the costs of contraceptives were aggregated, as well as studies that were economic evaluations of HCs for the management of other conditions such as heavy menstrual bleeding and emergency contraception were excluded. Details of the search strategy and keywords used can be found in Additional file 1. The search was limited to original research studies published in English prior to and including December 2019. Review articles, letters, editorials, commentaries and conference abstracts were excluded. Once duplicate citations were removed, all remaining titles and abstracts were screened by MS and categorized as being relevant, potentially relevant or not relevant. The potentially relevant full-text articles were then reviewed with AW and judged for relevance to be included in the registry. Additionally, hand-searching reference list of articles retrieved were screened to identify relevant economic evaluations. The relevant articles included complete economic evaluations namely, cost-effectiveness analyses (CEA), cost-utility analyses (CUA), cost-benefit analyses (CBA) and cost-minimization analysis (CMA). Partial economic evaluations included cost of illness or cost of unintended pregnancy (COUP), cost analyses (CA) and quality of life (QOL) studies. Studies classified as COUP provided country-wide estimates of the costs attributable to unintended pregnancy. The CA studies provided estimates of cost of the intervention without considering outcomes and the QOL studies measured health state utility values of unintended pregnancy. The Preferred Reporting Items for Systematic Reviews and Meta-Analysis (PRISMA) guidelines for developing literature search were followed.

### Variable selection and data dictionary

A list of variables to be included in the registry was developed after careful discussions with the expert panel. It was decided to match variables to the type of study (CEA, CUA, CBA, CMA, COUP, CA, QOL). The registry therefore included general bibliographic variables and information on the funding source, country and region. The variables extracted from studies included: 1) study objective; 2) intervention and comparator; 3) target population characteristics; 4) currency and year; 5) sample size; 6) perspective; 7) source of cost data; 8) direct cost components and amount; 9) non-medical cost components and amount; 10) indirect cost components and amounts; 11) outcome measure; 12) source of outcome data; 13) time horizon; 14) discounting and adjustment of costs; 15) scenario analysis; 16) modeling method description; 17) assumptions; 18) sensitivity analysis; 19) conclusions and 20) limitations. Variables from existing registries were used as guides in the variable development process [4–7]. Based on the type of economic study, other variables such as cost-benefit ratio, average cost-effectiveness (utility) ratio, incremental cost-effectiveness (utility) ratios, and quality-adjusted life-year (QALY) were catalogued. For COUP studies, we included variables to document the total number of unintended pregnancies and costs to the payer and impact of contraceptive non-adherence on total costs. During the course of data extraction of each article, if additional variables were found to be important for the registry, they were identified and included in the registry (e.g., variables for adjustment of cost of birth to account for mistimed rather than truly unwanted births). The data dictionary was continuously revised during this process to account for the changes and can be accessed in Additional file 2.

### Online data interface & data operationalization for querying

In order to facilitate entry of data into the registry by members of the expert panel and to collect the resulting data in real-time, an online data entry platform using Google Forms was created. However, to allow for querying the registry each variable was further operationalized, i.e., made either numeric (continuous or categorical) or a text entry (see data dictionary in Additional file 2).

### Expert panel verification process

The verification process included two-steps. After the expert panel members received assigned articles to critique, articles were entered into the registry using the Google Form. A formalized approach to review articles that were uploaded in the registry was used to ensure quality and consistency among all the reviewers. Responses collected were then transcribed in the operationalized database in Microsoft Excel by MS. Finally, AW verified all variables within each article. Discrepancies in entry were then discussed among AW and MS until a consensus could be agreed upon.

## Results

**Fig. 1:**
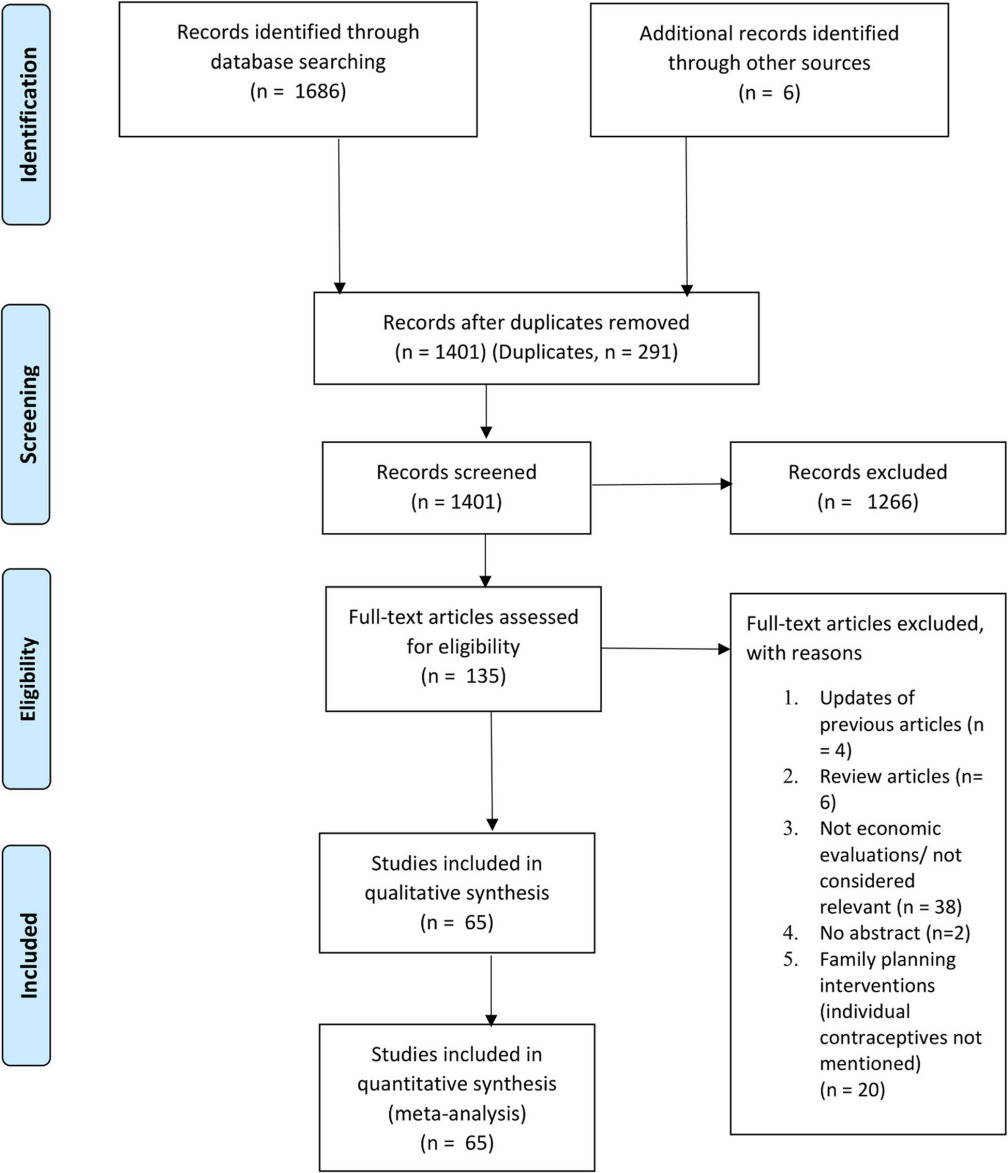
PRISMA flow diagram for the inclusion of studies in the pharmacoeconomic registry of hormonal contraceptives

From the 1401 articles that were retrieved from the literature search, excluding duplicates, the pharmacoeconomic registry included 65 articles comprising of 22 CEA, 9 CUA, 7 CBA, 1 CMA, 14 CA, 10 COUP and 2 QOL studies. The PRISMA flow diagram is shown in Fig. 1.

### Queries of the pharmacoeconomic registry

Most of the articles were published in the past 25 years except one from 1972 [22]. The complete registry can be accessed in Additional file 3. The table of cost and probabilities can be accessed in Additional file 4. Table 1 presents a summary of the CEA, CUA, CBA, CMA, CA and QOL studies. From the CEA and CUAs, ten studies were decision-tree models [27, 31, 38–40, 42, 48, 49, 51, 52] and eleven were Markov models [30, 32–34, 37, 41, 44–47, 50] and one was conducted using a systematic review and meta-analysis as part of a health technology assessment submission [28]. The remaining studies determined cost-effectiveness using real-world data or observational studies [22, 29, 35, 36, 43] or utilized a simple methodology considering costs and failure rates of each method [24–26] or a cost equation for clinical outcomes [23].

**Table 1:** Summary of economic evaluations and quality of life studies retrieved from HC pharmacoeconomic registry

	Author, year, country and reference	Objective	Interventions considered	Perspective	Time horizon	Cost effectiveness measure	Major finding
**Cost-Effectiveness Analysis**							
1	Kee, 1972 (Singapore) [22]	Cost-effect analysis of a family planning program	OCs, condoms, IUD	Singapore National Family Planning Program	3.5 years	Cost per birth prevented	Cost per birth prevented lowest for condom, followed by IUD and oral
2	Ashraf, 1994 (USA) [23]	Compare cost effectiveness of 8 methods	LNG implant, MPA injectables, OCs, copper-T IUD, vasectomy, tubal ligation, condom, diaphragm	Institutional	5,8 and 15 years	Net direct cost per pregnancy-free year	IUD most cost-effective, followed by LNG implants among reversible methods. Sterilization most cost-effective overall
3	Trussell, 1995 (USA) [24]	Compare effectiveness and costs of 15 contraceptive methods	Tubal ligation, vasectomy, OCs, implant, injectables, progesterone-T IUD, copper-T IUD, diaphragm, male condom, female condom, sponge, spermicides, cervical cap, withdrawal and periodic abstinence	Private and publicly funded payer	1 and 5 years	Total costs	Over 5 years, copper-T IUD was most cost-effective followed vasectomy, implant, and injectable
4	Hughes, 1996 (UK) [25]	Assess cost effectiveness of family planning services	OCs, injection, implant, IUD, condom, diaphragm, spermicide	National Health Service	1 year	Cost per pregnancy avoided, cost per CYP	IUD is most cost-effective followed by condom and implant
5	Trussell, 1997 (USA) [26]	Examine the cost and savings of contraceptive use in adolescent women compared with no method	Cervical cap, diaphragm, female condom, implant, injectable, male condom, OCs, periodic abstinence, spermicides, sponge, withdrawal, no method	Private and public payer	1 and 5 years	Total costs	All methods of contraception are cost-saving compared to no method. Extent of savings depend upon method
6	Phillips, 2000 (UK) [27]	Compare economic impact of long-acting reversible contraceptives	Implant (Implanon®), implant (Norplant®), LNG-IUS (Mirena®) and injectables (Depo-Provera®)	Payer	3 years (Implanon), 5 years (Mirena and Norplant)	Cost per pregnancy avoided	Implanon® more cost-effective than Norplant®, Mirena®, Depo Provera®
7	French, 2000 (UK) [28]	Estimate cost-effectiveness of implants and IUSs compared to other alternatives	Implant (Norplant®), LNG-IUS (Mirena®), Copper-T IUD, injectable (DMPA) and OC	NHS	1,2,3 and 5 years	Cost per pregnancy averted	Cost-effectiveness ratios for implants and IUSs quite high versus comparators, explained by low incremental effectiveness
8	Nakhaee, 2002 (Iran) [29]	Compare the cost-effectiveness of the seven methods and select the least costly way of providing a given level of contraceptive protection.	OCs, injectables, implants, IUD, tubal ligation, condom, vasectomy	Provider	Couple-year of protection	Cost per adjusted couple year of protection	Vasectomy, IUDs and oral contraceptives most cost-effective
9	Chiou, 2003 (USA) [30]	Examine the economic consequences of contraceptives available to women in the United States	LNG-IUS (Mirena®), Copper-T IUD, Injectable, OC, tubal ligation, diaphragm, spermicides, female condom, cervical cap	Third-party payer	5 years	Cost per average annual successful rate	LNG-IUS and copper-T IUD dominated over all methods except tubal ligation
10	Varney, 2004 (UK) [31]	Estimate the relative cost effectiveness of long-term hormonal contraception	LNG-IUS (Mirena®), Implant (Implanon®), MPA injectable (Depo-Provera®)	NHS	1 year	Annualized expected cost per expected annual number of pregnancies	LNG-IUS or implant are dominant compared to injectable
11	Sonnenberg, 2005 (USA) [32]	Quantify impact of increased adherence on the cost-effectiveness of the transdermal contraceptive patch in comparison to combination OCs	Transdermal patch, OC	Payer	2 years	Cost savings per pregnancies per woman	Patch is cost saving compared with OCs.
12	Mavranezouli, 2008 (UK) [33]	Assess cost-effectiveness of LARC methods that are used in the UK when compared to other contraceptive methods	Female sterilization, Implant, LNG-IUS, IUD, injectable (DMPA), OCs	NHS	1, 2, 3, 5, 10 and 15 years	Cost per average annual number of unintended pregnancies per 1000 women	LARCs dominated OCs. Female sterilization dominated LARC beyond 5 years. DMPA and LNG-IUS least cost-effective LARC
13	Trussell, 2009 (USA) [34]	Estimate the relative cost effectiveness of contraceptives in the United States	Copper-T IUD, vasectomy, LNG- IUS, male condom, fertility-awareness based methods, withdrawal, diaphragm, implant, spermicides, female condom, injectable contraceptive, sponge, tubal ligation, vaginal ring, OC, transdermal patch	Payer	5 years	Five-year cost per average annual rate of not becoming pregnant over 5 years	Copper-T IUD, vasectomy and LNG-IUS are the most cost-effective methods
14	Lipetz, 2009 (UK) [35]	Compare the cost-effectiveness of Implanon in comparison to OCs in a community setting	Implant (Implanon®), oral contraceptives	Payer	1,2 and 3 years	Cost per patient per year of use (outcomes included)	Implanon® is more cost-effective than OCs at all time points
15	Ames, 2012 (Canada) [36]	Determine if provision of free IUDs postabortion is associated with a reduction in health-care costs and repeat abortions compared with provision of OCs or DMPA.	Copper-T IUD, OC, injectable, condom	Payer	5 years	Total cost per woman for contraception and repeat abortions per repeat abortion rates (1 and 5 years)	Immediate insertion of IUDs postabortion associated with lower 5-year rate of repeat abortion and cost reduction versus OCs or DMPA
16	Trussell, 2014 (USA) [37]	Evaluate the cost-effectiveness of LNG-IUS 13.5 mg in comparison with SARC methods in a cohort of young women in the US	LNG-IUS 13.5 mg, SARC mixed basket (branded and generic oral contraceptives, ring, patch and injections)	Third-part payer	3 years	Cost per unintended pregnancies avoided	LNG-IUS 13.5 mg is cost-effective compared to SARC
17	Han, 2014 (USA) [38]	Determine the cost-effectiveness of a hypothetical state-funded program offering immediate postpartum implant (IPI) insertion for adolescent mothers.	Immediate postpartum subdermal implant insertion, standard contraceptive initiation	Colorado Medicaid	6,12,24,36 months	Costs saved per repeat pregnancy rate	At 12, 24 and 36 months, offering IPI is cost-effective
18	Heitmann, 2014 (USA) [39]	Estimate the number of unintentional pregnancies in active duty women that could be prevented annually by the use of a LNG-IUS and direct cost savings	LNG-IUS (Mirena®)	US government health care system	1 year	Cost per number of unintended pregnancy	Use of LNG-IUS could result in significant reductions in unintended pregnancies resulting in cost savings.
19	Gariepy, 2015 (USA) [40]	Evaluate the cost-effectiveness of immediate compared with delayed (6 weeks) postpartum etonogestrel implant insertion in preventing future unintended pregnancy.	Immediate insertion of implant (after delivery but before discharge), Delayed postpartum implant insertion (e.g, at 6 week postpartum visit)	Payer	1 year	Cost per expected pregnancy rate	Immediate postpartum contraceptive implant is cost-effective in preventing unintended pregnancies
20	Trussell, 2015 (USA) [41]	Estimate the average annual cost of available reversible contraceptive methods in the United States and quantify minimum duration of use required for LARC methods to achieve cost-neutrality relative to other reversible contraceptive methods while taking into consideration discontinuation.	Copper IUD, implant, LNG-IUS, generic OC, ring, patch, injection, mixed-SARC, condom	Payer	1,2,3,4,5 years	Annualized costs by year, per woman (outcome included)	Copper-IUD and LNG-IUS were the least expensive methods. LARC methods become cost-saving relative to SARC methods within 3 years of usage even if they are not used for their full duration of efficacy.
21	Canestaro, 2017 (USA) [42]	Estimate the relative cost effectiveness of insurance coverage of contraception under employer-sponsored insurance coverage taking into consideration newer regulations allowing for religious exemptions.	Full contraceptive coverage through an employer-sponsored private health insurance plan (OCs, tubal ligation, IUD, injectable, vaginal ring, transdermal patch, implant) versus no contraceptive coverage	Employer	1 year	Costs per woman, number of unintended pregnancies	Not providing contraception coverage resulted in greater number of unintended pregnancies resulting in higher total costs among uninsured women.
22	Agostini, 2018 (France) [43]	Assess the effectiveness and costs associated with contraceptive methods based on real-world data in France	1st-2nd generation combined OCs, 3rd generation combined OCs, progestin-only pill, copper-IUD, LNG-IUS, etonogestrel implant	Health system	2 years	costs including unplanned pregnancies cost	LARCs should be considered for a broader use to prevent unplanned pregnancies.
**Cost-Utility Analysis**							
1	Sonnenberg, 2004 (USA) [44]	Determine the costs and net health effects of various methods of contraception	Vasectomy, injectable (DMPA), copper-T IUD, LNG-IUD, patch, vaginal ring, OCs, monthly injectable, periodic abstinence, withdrawal, diaphragm, tubal sterilization, no method	Societal	2 years	Cost per QALY	All contraceptive methods result in substantial cost-saving compared to no use of contraception. Vasectomy resulted in highest cost-savings followed by DMPA, copper-IUD and LNG-IUD
2	Babigumira, 2012 (Uganda) [45]	Compare the incremental cost-effectiveness of a hypothetical new contraceptive program that would achieve universal access to modern contraceptives in Uganda, to the current contraceptive program	New contraceptive program (universal access to modern contraceptives in Uganda), current contraceptive program (status quo in which access to modern contraception is limited)	Societal and governmental	Lifetime	Cost per DALY averted, cost per life-year gained, cost per pregnancy averted, cost per unit of fertility reduction	Universal access to modern contraceptives dominated the current contraceptive program and is highly cost-effective.
3	Burlone, 2013 (USA) [46]	Model the cost-effectiveness of expanding contraceptive coverage from 185 to 399% FPL for insurance exchange plan providers in Oregon, as it examines the impact of expanded coverage of currently uninsured women in Oregon.	Increase contraceptive coverage to < 399% FPL versus Maintaining contraceptive coverage at < 185% FPL	Oregon state insurance plan providers	5 years	Cost per number of pregnancies and QALY	Extending contraceptive coverage under the Affordable Care Act is cost-saving and cost-effective
4	Henry, 2015 (Sweden) [47]	Evaluate the cost-effectiveness LNG-IUS 13.5 mg (Jaydess®) vs. OC, in women at risk of unintended pregnancy.	LNG-IUS 13.5 mg, oral contraceptive, LNG-IUS (Mirena®), Hormonal market mix of methods	Societal	3 years	Cost per unintended pregnancy avoided and cost per QALY	LNG-IUS 13.5 mg is generated cost-savings and resulted in fewer unintended pregnancies compared with OCs
5	Washington, 2015 (USA) [48]	Determine if immediate postpartum IUD placement prevents pregnancy and is cost-effective compared with routine placement.	Immediate postpartum IUD placement (within 10 min of placental expulsion), routine IUD placement (6–8 weeks postpartum)	Health care	2 years	Cost per total number of unintended pregnancies and cost per QALY	Immediate postpartum IUD is a dominant strategy over routine IUD placement
6	Di Giorgio, 2018 (Uganda) [49]	Assess the cost-effectiveness of self-injected subcutaneous DMPA-SC compared to health-worker-administered intramuscular DMPA (DMPA-IM)	Self-injected subcutaneous DMPA-SC, Health-worker-administered intramuscular DMPA (DMPA-IM)	Societal and health system	1 year	Costs per pregnancies averted, Costs per DALY averted	Under a societal perspective, self-injected DMPA-SC averted more pregnancies and was cost-saving compared to health worker administered DMPA-IM
7	Gumbie 2019 (Australia) [50]	Estimate cost-effectiveness of reclassifying OCs from prescription to pharmacist-only	Prescription-only OCs, pharmacist-only OCs	Healthcare system	35 years	Cost per QALY	Reclassifying OCs from prescription only to pharmacist-only was more effective and cost saving
8	Rodriguez 2019 (USA) [51]	Estimate unintended pregnancies averted and cost-effectiveness of pharmacist prescription of hormonal contraception	With and without pharmacist prescription of hormonal contraception	Payer (Oregon Medicaid)	1 year	Unintended pregnancies averted, costs and QALYs	Policy expanding scope of pharmacist to prescribe hormonal contraception averts unintended pregnancies and is cost effective
9	Mvundura 2019 (Senegal) [52]	Evaluate the cost-effectiveness of self-injected subcutaneous DMPA-SC compared to health-worker-administered intramuscular DMPA (DMPA-IM)	Self-injected subcutaneous DMPA-SC, Health-worker-administered intramuscular DMPA (DMPA-IM)	Societal and health system	1 year	Costs per DALY averted	Under a societal perspective, self-injected DMPA-SC averted more pregnancies and cost less compared to health-worker administered DMPA-IM
**Cost-benefit analysis**							
1	Ortmeier,1994 (USA) [53]	Examine the net benefit of four hormonal methods of contraception based on the costs and benefits per patient per day of effective pregnancy prevention	Injectable (DMPA), implant (Norplant®), progestogen only pill (Nor-QD®), combined pill (Ortho-Novum 7/7/7®)	Payer (Managed care)	1 patient-day	Net benefit per patient per day	All methods have a positive net benefit. DMPA shows highest net benefit, followed by Nor-QD® and Ortho-Novum 7/7/7®).
2	Foster, 2009 (USA) [54]	Assess the cost-effectiveness of contraceptive methods dispensed in 2003 for 955,000 women in Family PACT Program- California’s publicly funded family planning program.	Interval tubal ligation, implant, IUD, injectable, ring, patch, OCs, barrier methods, emergency contraceptives	Payer (Family PACT California’s publicly funded family planning program)	Varies depending upon contraceptive method	Cost-savings per dollar expenditure	Implant was most cost-effective followed by IUD, injectables, OCs and patch
3	Rodriguez, 2010 (USA) [55]	Examine the hospital and state costs of offering the option of a postpartum IUD to an underinsured population of recent immigrants to the United States with Emergency Medicaid (EM) insurance coverage only	Postpartum intrauterine device (IUD) versus absence of program	Hospital and state	1,2,3,4 years	Cost-savings per dollar expenditure	Postpartum IUD is cost beneficial from state government perspective but not from hospital perspective
4	Onwujekwe, 2013 (Nigeria) [56]	Determine the willingness to pay (WTP) and the benefit-cost of modern contraceptives delivered through the public sector in Nigeria.	Male condom, female condom, OC, injectable, implant, IUD	Public-sector payer	1 year	Unit price (cost) per mean WTP amount (benefit)	The benefits of providing contraceptives through public sector far outweighed the costs, except for female condoms
5	Foster, 2013 (USA) [57]	Examine relative cost-benefit of specific methods and evaluate the relative contribution of each method to the number of unintended pregnancies averted within the Family PACT population.	Tubal ligation, tubal occlusion, copper-IUC, hormonal IUC, implant, injectable, ring, patch, OC, barrier methods, emergency contraceptives	Payer (Family PACT California’s publicly funded family planning program)	Varies depending upon contraceptive method	Cost savings per dollar expenditure	Copper-IUC was most cost-saving followed by implant and hormonal IUC.
6	Keen, 2017 (Sierra Leone) [58]	Estimate the costs and benefits of scaling up family planning in Sierra Leone.	Pill, condom, injectable, IUD, implant, female sterilization, male sterilization, lactational amenorrhea	Payer	5, 12 and 22 years	Cost-savings per dollar expenditure	Every dollar spent on family planning is expected to save US$2.10 in expenditure on selected social sector services
7	Concepcion, 2019 (Australia) [59]	Evaluate economic effect of an increase in LARC uptake to international rates in Australia	LARC methods- etonogestrel implant, copper IUD and hormone releasing IUS	Government and consumers	5 years	Cost savings per woman per year, cost savings over 5 years	Greater use of LARC would result in net gains in economic benefits to Australia
**Cost-minimization analysis**							
1	Wilkinson, 2019 (USA) [60]	Analyze Indiana Medicaid’s cost-savings associated with providing adolescents with same-day access to LARC	Same day access to LARCs, subsequent visit for LARC placement	Payers (Medicaid)	1 year	Costs, rates of unintended pregnancy and abortion	Providing same-day LARC was cost-saving and associated with lower pregnancy and abortion rate
**Cost-analysis**							
1	Janowitz, 1994 (Thailand) [61]	Study the impact of providing implants on method use and costs	Implant, IUD and injectable	Thailand’s National Family Planning Program	1, 2, 3, 3.5 and 5 CYP	Cost per CYP	Cost per CYP was higher for implant than for IUD or injectables
2	Koenig, 1996 (USA) [62]	Measure the social costs associated with selected contraceptive methods, comparing them with each other and with the use of no method.	Copper-T IUD, diaphragm, implant, injectable, male condoms, OC, tubal ligation	Social welfare programs and Medicaid	5 years	Total social welfare and direct medical savings from contraceptives	Copper-T IUD followed by implant and oral contraceptives resulted in the greatest social welfare and direct medical savings
3	Margulies, 2001 (USA) [63]	Measure use rates of DMPA and OC and compare costs between them to see whether these trends impacted pharmaceutical acquisition costs for a family planning program over three time periods (1992, 1994 and 1999)	DMPA, OC	Pharmacy family planning budget	3 years (1992, 1994 and 1999)	Costs	High cost of DMPA (due to non-availability of generic or competing product) could jeopardize pharmacy to offer this method to women
4	Lipetz, 2009 (UK) [64]	Determine how long clients were keeping their contraceptive implants in and cost of implant provision	Implant (Implanon®)	Community based sexual and reproductive health service	1 year	Costs	The annual cost for using Implanon® was 25% lower than the estimate made by NICE despite a shorter duration of use
5	Tumlinson, 2011 (Kenya) [65]	Assess whether implant clients in Kenya are paying as much or more than the direct service delivery cost of Sino-implant (II)	Sino-implant (II)	Patient	1 year	Costs	Patient fees in private sectors allow for 100% recovery of direct cost of providing Sino-implant (II), therefore potential to reduce reliance on donor-supplied implants thereby improving contraceptive security
6	Chin-Quee, 2013 (Zambia) [66]	To determine the incremental cost per CYP of adding injectable contraceptives to the existing community health worker (CHW) family planning program	Adding injectable contraception (DMPA) into existing community health worker’s family planning program	NA	1 year	Cost per CYP	Provision of injectable contraceptives by CHW is safe, acceptable and feasible in Zambia with high rates of uptake in hard-to-reach areas.
7	Salcedo, 2013 (USA) [67]	Evaluate potential cost savings associated with immediate postabortal IUD insertion compared with planned IUD insertion at time of abortion follow up	Immediate postabortal IUD insertion, planned IUD insertion at time of abortion follow up	Public payer	1, 5 year	Cost savings per woman	Immediate postabortal IUD insertion is cost saving compared to planned IUD insertion at time of abortion follow up
8	Cook, 2014 (UK) [68]	Establish the actual costs of providing the IUS in a community sexual and reproductive health setting and compare it to the cost predicted by NICE	Intrauterine system	Community based sexual and reproductive health service	1 year	Cost per patient per year	Providing IUS in community clinics was 23% cheaper than that predicted by NICE and cheaper than providing combined OCs
9	Schnippel, 2015 (South Africa) [69]	Conduct a cost evaluation of establishing a van-based mobile clinic in two rural districts in South Africa that provider cervical cancer screening and other reproductive and primary health services	OC, norethisterone enanthate (injectable), MPA (Injectable), male condoms, female condom	Health service provider	1 year	Unit cost per patient	Staffing costs are the largest component of providing mobile health services to rural communities.
10	Chola, 2015 (South Africa) [70]	Estimate the service delivery cost of scaling up modern contraception, and potential impact on maternal, newborn and child survival	Male condom, female sterilization, male sterilization, injectable contraceptive, Implanon®, OC, IUD	Health service provider	16 years	Unit costs of contraceptive methods per year, total annual cost of family planning for South Africa	Scaling up family planning can have huge impacts on maternal and child mortality
11	Foster, 2015 (USA) [71]	Estimation of how making OCs available without a prescription may affect contraceptive use, unintended pregnancies and contraceptive and pregnancy costs among low-income women	OTC OC access, no OTC access to OCPs	Public sector costs	1 year	Costs per woman	If out-of-pocket costs for OCs are low, OTC access could increase use of effective contraceptives and reduce unintended pregnancies
12	Rademacher, 2016 (Kenya) [72]	Calculate direct service delivery cost per CYP of various family planning methods	Copper-IUD, male sterilization, female sterilization, male condom, Jadelle® implant, Sino-implant (II), Implanon® implant, LNG-IUS, OCs, DMPA injectable, Sayana Press® injectable, NET-EN injectable, female condom	NA	CYP	Costs per CYP	Introduction of LNG-IUS has the potential to increase access and choice for women in Kenya.
13	Law, 2017 (USA) [73]	Evaluate differences in mean costs per woman of the use of two IUDS	Mirena® and Liletta®	Payer	3, 5 and 10 years	Costs per woman	Mirena® was associated with slightly higher cost than Liletta® at 3 years, but was more cost-saving at 5 and 10 years
14	Madden, 2018 (USA) [74]	Conduct a cost-savings analysis of Contraceptive CHOICE Project, which provided counseling and no-cost contraception, to demonstrate value of investment in enhanced contraceptive care	IUD, implant, injectable, OCs, patch, ring, natural family planning, male condom, no method	Missouri Medicaid	45 months	Total costs for contraceptive CHOICE project and simulated comparison group	Providing no-cost contraception results in substantial cost-savings because of increased uptake of highly effective contraception and averted unintended pregnancy and birth
**Quality of life studies**							
1	Schwarz, 2008 (USA) [75]	Assess the potential impact of unintended pregnancy on women’s quality of life	NA	Non-pregnant women	NA	VAS, TTO, SG metrics of health state utility values for unintended pregnancy	Provided estimates on the anticipated effects of pregnancy on women’s quality of life to be integrated into CEAs
2	Lundsberg, 2017 (USA) [76]	Contribute to decision analysis by estimating utility for different pregnancy contexts	NA	Pregnant women	NA	VAS, TTO, SG, PROMIS GSF-derived utility	Unintended pregnancy is associated with significant disutility.

*OCs* Oral contraceptives, *IUD* Intra uterine device, *LNG* Levonorgestrel, *MPA* Medroxyprogesterone acetate, *CYP* Couple year of protection, *DMPA* Depo-medroxyprogesterone acetate, *IUS* Intra-uterine system, *LARC* Long-acting reversible contraceptive, *SARC* Short-acting reversible contraceptive, *QALY* Quality-adjusted life-year, *DALY* Disability-adjusted life-year, *FPL* Federal poverty level, *IM* Intra-muscular, *SC* Subcutaneous, *NICE* National Institute for Health and Care Excellence, *NHS* National Health Service, *OTC* Over the counter, *NET-EN* Norethisterone enanthate, *VAS* Visual analog scale, *TTO* Time trade-off, *SG* Standard gamble

Among all the CEA, CUA, CBA, CMA, CA and QOL studies, 29 were conducted among the US population, eight in the UK, two each in Australia, Kenya, South Africa, Uganda and one each from Canada, France, Iran, Nigeria, Senegal, Sierra Leone, Sweden, Thailand, Singapore and Zambia (Table 1). Based on the country-wise distribution, in the published literature there were few economic evaluations conducted in developing countries as compared to developed countries.

While most studies compared the cost-effectiveness of different methods of contraception, some studies addressed more specific issues (Table 1). Seven studies compared the impact of immediate postpartum or post-abortion insertion of long-acting reversible contraceptives (LARC) with delayed insertion [36, 38, 40, 48, 55, 60, 67]. Three studies examined the cost-savings from increasing access to oral contraceptives by making available over-the-counter (i.e., without a prescription) or allowing pharmacists to prescribe them [50, 51, 71]. Ten studies considered the impact of enhancing access to contraceptives in Africa utilizing novel delivery methods such as community health workers and self-injection [45, 49, 52, 56, 58, 65, 66, 69, 70, 72]. Among the studies conducted in US, several focused on populations that faced disparities in access to contraceptives. The majority of studies focused on Medicaid-eligible or low-income women [24, 26, 38, 40, 51, 54, 55, 57, 60, 62, 63, 67, 71, 74]. Three studies considered adolescent contraceptive provision [26, 38, 60] and one focused on active-duty military women [39]. Two studies considered the underinsured, quantifying the impact of expansion of contraceptive coverage through Affordable Care Act (ACA) [42, 46].

Except for one CBA that determined the willingness-to-pay for contraception [56], all studies considered direct medical costs in their analyses. Direct non-medical costs were considered among six studies [22, 29, 45, 49, 52, 62] and included overhead costs and capital costs, utilization of auxiliary services and social welfare program costs. Indirect costs were considered in eleven studies and included waiting and travel times, productivity losses and personnel training times [22, 35, 45, 47, 49, 52, 64, 70, 77–79]. No study considered the impact of intangible costs. Fifteen studies considered the cost of side effects, out of which seven studies mentioned disaggregated costs of each side effect [23, 24, 30, 32, 34, 44, 50].

Quality of life, in the form of QALY was considered in six of the nine CUAs with the remaining considering DALYs averted (Table 1) [45, 49, 52]. Methodological improvements have occurred over time in the conduct of CUAs, especially with regards to the source of health utility values. In the absence of published estimates, the earliest CUA employed utility values for pregnancy outcomes and other health states that were elicited from a convenience sample of female members of the research team and an advisory panel and these estimates were used in a later CUA [44, 48]. Recognizing the paucity of reliable utility values for unintended pregnancy, Schwarz et al. [75] conducted a study to assess the potential impact of unintended pregnancy on non-pregnant women. These values were used in subsequently conducted CUAs [46, 47, 50, 51]. In an effort to supplement future CUAs in pregnancy and contraception, Lundsberg et al. measured utility values from a sample of pregnant women for varying contexts such as intention, timing and wantedness (Table 1) [76]. However, the literature is lacking utility values of various pregnancy contexts and outcomes from developing country settings.

Table 2 summarizes the COUP studies. Most of these studies provided country-wide estimates of the burden of unintended pregnancies in the USA [80, 81], Norway [77], Sweden [78], UK [82], Spain [83], South Africa [79], Canada [84] and Russia [85], however, one study estimated these costs for a US employer-sponsored health insurance plan [86]. Imperfect contraceptive adherence contributed to over half of these costs, thus making a case for greater LARC adoption [77, 78, 80, 83, 84].

**Table 2:** Cost of unintended pregnancies by country

Author, year, country and reference	Perspective	Cost of unintended pregnancy, millions (US$, 2020)^**b**^	% attributed to imperfect adherence
Trussell, 2013 (USA) [80]	Third party health care payer	5426	53%
Trussell, 2007 (USA) [81]	Payer	7056	NA
Henry, 2015 (Norway) [77]	Societal	42	60.40%
Engstrand, 2018 (Sweden) [78]	Societal	117	61.40%
Montouchet, 2013 (UK) [82]	NHS	330	NA
Lete, 2015 (Spain) [83]	Spanish National Health System	483	69%
Le, 2015 (South Africa) [79]	Payer	675^a^	NA
Black, 2015 (Canada) [84]	Public payer	293	69%
Lowin, 2015 (Russia) [85]	Generic payer	898	NA
Dieguez, 2015 (USA) [86]	Employer-Sponsored health insurance plans	NA	NA

*NHS* National Health Service NA signifies values not mentioned in study. ^a^Costs not adjusted for mistimed pregnancies. After adjustment costs likely to be lower. ^b^Direct costs mentioned for all except Norway and Sweden which includes indirect costs. All costs converted to 2020 USD using IMF derived PPP values using CCEMG-EPPI- Centre Cost Converter (v.1.6) https://eppi.ioe.ac.uk/costconversion/default.aspx

### Purposes of pharmacoeconomic registry and best practice recommendations

The completed HC pharmacoeconomic registry can serve various purposes to guide future research and policy as mentioned in Table 3. We have used cost implications of DDIs with HCs as an example to demonstrate how the registry could be used to inform this decision problem. This project was undertaken to systematically document the key variables in all published pharmacoeconomic literature pertaining to HCs. Inclusion of all types of economic articles, including QOL allowed for the creation of an up-to-date compendium, that could aid future researchers and funding agencies in identifying existing research and prioritize future research. Costs, probabilities and utility values documented from each study, including their original references, allows for the collection of input variables for subsequent pharmacoeconomic analyses and budget impact models. Additionally, the registry catalogues the various published model structures, methodologies and assumptions which inform the development of subsequent models.

**Table 3:** Various purposes for developing a pharmacoeconomic clinical registry and how querying the registry can inform decision making

Purposes	Example using HC pharmacoeconomic registry	Informing decision-making in HC DDIs
1. Helps the researcher understand the evolution of PE literature in a specified topic	The earliest literature evaluating the PE of contraception was published in 1972. While being a crude analysis from modern PE literature standards, it provides useful information regarding the cost effectiveness of contraceptives available at that time. Since the mid 1990’s the PE literature in this area has been steadily increasing.	The variability in the conduct of these PE analyses of HCs over the years provides guidance on how future CEAs need to be conducted
2. To evaluate trends, compare published models and identify gaps in the literature to guide future research	Given the impact of underlying disease on contraceptive failure, for example, the potential for DDIs, very few studies considered contraceptive use in special populations. There is also a lack of data on the impact of HC failure on QOL	Provides decision-makers, funding agencies and researchers with evidence regarding potential areas of future research focus and the need to generate this data along with PE studies in developing regions.
3. Provides a systematic approach to evidence collection to build PE models, especially when published data is sparse.	PE evidence pertaining to the cost implications of DDIs with HCs is lacking in the literature. The registry provides a quick and ready access to data sources that can provide the inputs to develop a PE model to address this question which could help in clinical guideline recommendations for practitioners and decision makers.	Quantifying the cost implications of DDIs with HCs, along with clinical data can provide regulatory agencies with an estimate of the magnitude of the problem
4. Data obtained can be used to perform systematic reviews of economic evaluations and meta-analysis	A systematic review of economic evaluations can be performed using the articles retrieved to generate more robust evidence.	Systematic reviews of economic evaluations can help critically evaluate studies for their methodological and reporting quality, thus guiding future studies in the field.
5. Helps evaluate economic literature from a global perspective to guide policy decisions	There is a lack in studies conducted in developing countries, where the disease burden of drugs involved in DDIs (such as tuberculosis and HIV medication) is high. Evidence from burden of illness and cost analysis conducted in developing countries can be used to supplement the existing evidence and develop budget impact models.	Understanding the landscape of the target population (women at risk of DDIs with HCs) in various countries can inform policies on optimal contraceptive choice in these populations

*PE* Pharmacoeconomic, *DDI* Drug-drug interactions

Developing the registry in an iterative manner and using a trial-and-error approach allowed us to identify some of the methodological best practices for the development of a pharmacoeconomic registry which are summarized in Table 4. We recognized the importance of dissemination and transparency of the registry and therefore decided to make it open-access to allow researchers to further improve and use the registry for their own research. Therefore, early planning and engagement with project collaborators, funding agencies, and journals should be undertaken to ensure open availability of the registry. The registry should be well documented by defining each variable in columns and how to read data across rows using an accompanying data dictionary. A core team comprising of experts in fields such as pharmacy, pharmacoeconomics and outcomes research must be assembled to provide continuous feedback on the development and validity of the registry. Variables extracted should be operationalized to allow for querying to the extent desired. Finally, the database must be extensively queried to identify potential transcription errors and areas for improvement. For example, a testing exercise may reveal that a new variable may need to be added in the registry, in that case, the change has to be then made for all prior entered studies.

**Table 4:** Methodological best practices and associated considerations for developing a pharmacoeconomic clinical registry

Best practices	Considerations	Need
1. *Transparency, public availability and dissemination:* Enable provision of registry to the public with minimal barriers to access.	• Planning ahead to discuss with funding agency, other stakeholders about making the proposed registry publicly available upon completion • Should use universally used software to access (e.g. Microsoft Excel) • Must have clearly defined documentation accompanying the registry to aid in defining terms and usability.	• Promotes greater collaboration, especially among policy-makers in low and middle income countries • Crowd-sourcing of potential studies for inclusion into the registry • Identification and correction of potential errors or incorrect information
2. *Expert panel:* Convene a team of experts in pharmacoeconomics and outcomes research	• Include a team consisting of a trainee along with several PhD-level experts • Experts should have the time and willingness to aid in the review, data extraction and update meetings	• A team-based approach to curating data for a registry ensures accuracy and allows for provision of continuous feedback
3. *Data operationalization:* Ensure that the registry is queryable to the extent desired	• Developers of a registry can start by having descriptive variable fields which can then be operationalized as categorical to enhance filtering and querying capabilities	• Variables that are coded as nominal or ordinal categories can aid in data analysis
4. *Testing:* Provide access to beta version of the registry to various stakeholders at regular intervals to gather additional feedback	• Setting the variables of the registry a priori results in lesser potential to account for future changes and improvements to the registry • Identify overt transcription errors	• Flexibility and iteration in registry building process allows for customizing registry for the purpose of the individual study

## Discussion

The article presents the development of a pharmacoeconomic registry using a case study of economic evaluations and QOL studies conducted on HCs for the purpose of contraception. The best practice recommendations for curating this registry and various cases for use are also presented. The registry included 65 studies, covering a variety of contraceptive interventions across various countries and settings.

One of the major questions that guided the development process was, “How would a potential researcher utilize this resource?”. Therefore, emphasis was placed on ensuring transparency, open availability and wide dissemination throughout the process. Building credibility into the resource requires documenting every step undertaken to enable reproducibility of the final outcome [87]. In this regard, there are two questions worth considering: 1) Was the original study described in a transparent manner? and 2) Does the registry adequately reflect the information contained within the study? In order to address the first question, while standard quality assessment checklists and reporting standards such as the consolidated health economic evaluation reporting standards (CHEERS) guided our variable selection process, we did not formally assign a score for each study [88]. This is because, our registry was not restricted to full economic evaluations, but also included partial economic analysis such as CAs and cost of illness studies (COUP), elements of which may not be fully met by criteria mentioned in quality assessment checklists. Additionally, while all the studies essentially aimed to answer the question of the cost-effectiveness or savings that access to contraceptives can provide in various settings, the methodology and outcome measure used varied considerably among the studies, especially those conducted pre-2000 (Table 1). We, therefore, leave it to the researcher to decide whether estimates or conclusions derived from a particular study from the registry should be weighted differently as compared to another study for their individual research purposes.

Other well-known registries are good resources which document economic evaluations conducted on non-disease specific interventions [4, 5, 7]. A query using the term “contraceptive” on the Tufts CEA registry showed 15 studies, of which four were on contraceptives used for contraception, with the other studies focused on contraceptives used for other clinical conditions such as uterine fibroids, endometriosis and dysmenorrhea. The Tufts DALY study revealed one study and the NHS EED had 50 studies of which 20 were relevant to our clinical question. However, the Tufts CEA registry is not completely publicly accessible, and the NHS EED has discontinued updates and lacks a querying functionality. Therefore, we decided to create this specific, open-access database that can be queried. This is also in response to calls from the health economics community advocating for greater transparency and openness in health economic evaluations [11, 89–93]. The features of our registry seem to address the barriers to utilization of cost-effectiveness models and meet the desirable features of such a database as assessed by Teerawattananon et al. during interviews of policymakers, technical advisors and researchers in Bangladesh, India and Vietnam [94].

Our registry offers a resource for researchers working in the field of family planning and reproductive health, which is an important component towards fulfilling the United Nations sustainable development goal 3 [95]. It was therefore not aimed at synthesizing information from methodologically similar studies, as is common in systematic reviews, rather the registry aimed to provide an all-encompassing overview of economic studies conducted among various methodologies. Systematic reviews on economic evaluations of contraceptives have been conducted by Mavranezouli [96] and Lynch et al. [97], with one considering family planning interventions in general in low and middle income countries conducted by Zakiyah et al. [98] Economic evaluations addressing contraceptives especially in developing country settings is lacking [96]. This remains an important challenge, since results of economic evaluations from developed countries may not be transferable to developing country settings, given the vastly different payment models (payer versus out of pocket) and contraceptive use patterns, among other differences [19, 99]. While the number of studies conducted in the US was the most among other countries in our registry, a recent analysis revealed a lack of cost-utility analysis addressing the Healthy People 2020 priority areas, which includes reproductive and sexual health [100].

Our study had some limitations that can be addressed in future iterations of the registry. The CAs that were retrieved for the registry were only those identified from the database literature search, hand-searching was not extensively conducted to identify other cost analyses. We did not include budget impact analyses and cost-consequences analyses in our registry. Other sources from which economic studies obtain their probabilities such as clinical trials or observational studies, or reports from the Guttmacher Institute [16], UN [19] and National Survey of Family Growth [101] were not included as they were not in the scope of our current registry.

## ConclusionGliklich RE, Dreyer NA, Leavy MB. Registries for evaluating patient outcomes: a user’s guide. 3rd ed. Rockville (MD): Agency for

The current paper provides a description of the development of a pharmacoeconomic clinical registry using a case study of economic evaluations conducted on hormonal contraceptives. The registry is a comprehensive database which provides key variables from all published economic literature relating to HCs and is hoped to serve as a tool that could aid in identifying global trends and avenues for future research and economic models. The development of best practices to appropriately design and execute a pharmacoeconomic clinical registry can provide helpful information to researchers and clinical practitioners for comparative effectiveness decision making. We invite the health economics community to make use of this resource for their own research purposes.

## Supplementary Information

**Additional file 1.**

**Additional file 2.**

**Additional file 3.**

**Additional file 4.**

## Data Availability

The datasets supporting the conclusions of this article and associated files are included within the article as supplementary information files.

## Abbreviations

QOL: Quality of life
RCT: Randomized control trials
DALY: Disability-Adjusted Life Year
QALY: Quality-adjusted life-year
NHS EED: National Health Service (NHS) Economic Evaluations Database
EURONHEED: European Network of Health Economic Evaluation Databases
WHO-CHOICE: WHO Cost-Effectiveness and Strategic Planning
HC: Hormonal contraceptives
DDI: Drug-drug interactions
CEA: Cost-effectiveness analyses,
CUA: Cost-utility analyses
CBA: Cost-benefit analyses
CMA: Cost-minimization analysis
COUP: Cost of unintended pregnancy
CA: Cost analyses
PRISMA: Preferred Reporting Items for Systematic Reviews and Meta-Analysis
LARC: Long-acting reversible contraceptives
ACA: Affordable Care Act
OCs: Oral contraceptives
IUD: Intra uterine device
LNG: Levonorgestrel
MPA: Medroxyprogesterone acetate
CYP: Couple year of protection
DMPA: Depo-medroxyprogesterone acetate
IUS: Intra-uterine system
SARC: Short-acting reversible contraceptive
FPL: Federal poverty level
IM: Intra-muscular
SC: Subcutaneous
NICE: National Institute for Health and Care Excellence
NHS: National Health Service
OTC: Over the counter
NET-EN: Norethisterone enanthate
VAS: Visual analog scale
TTO: Time trade-off
SG: Standard gamble
