# Supplementary material for: Development of a pharmacoeconomic registry: an example using hormonal contraceptives
Source: Health Econ Rev. 2021 Mar 20;11:10. doi: 10.1186/s13561-021-00309-z (PMC7981865; doi:10.1186/s13561-021-00309-z)
Supplement: Supplementary file 1 — Additional file 1. [file 13561_2021_309_MOESM1_ESM.docx]

| Description | S.no | Strategy |
| --- | --- | --- |
| Indication | 1 | oral contraceptives[MeSH Terms] |
|  | 2 | hormonal oral contraceptives[MeSH Terms] |
|  | 3 | "Agents, Estrogen Contraceptive" OR "Contraception" OR "Contraceptive Agents, Estrogen" OR "Contraceptive Agents, Oral, Hormonal" OR "Contraceptives, Hormonal Oral" OR "Contraceptives, Low-Dose Oral" OR "Contraceptives, Phasic Oral" OR "Estrogen Contraceptive Agents" OR "Hormonal Oral Contraceptive Agents" OR "Hormonal Oral Contraceptives" OR "Low-Dose Oral Contraceptives" OR "Oral Contraceptive Agents, Hormonal" OR "Oral Contraceptives" OR "Oral Contraceptives, Hormonal" OR "Oral Contraceptives, Low Dose" OR "Oral Contraceptives, Low-Dose" OR "Oral Contraceptives, Phasic" OR "Phasic Oral Contraceptives" |
|  | 4 | "Hormonal contraceptives" OR "Hormonal contraception" OR "Contraceptives, hormonal" |
|  | 5 | #1 OR #2 OR #3 OR #4 |
| Medications | 6 | "l-Norgestrel" OR "l Norgestrel" OR "D-Norgestrel" OR "D Norgestrel" OR "Microval" OR "duofem" OR "Microlut" OR "Mirena" OR "Norgeston" OR "Norplant-2" OR "Norplant 2" OR "Norplant2" OR "NorLevo" OR "Norplant" OR "Plan B" OR "Vikela" OR "Cerazet" OR "Capronor" OR "levonorgestrel" |
|  | 7 | "Ethinyl Estradiol" OR "Estradiol, Ethinyl" OR "Ethinyloestradiol" OR "Ethynyl Estradiol" OR "Estradiol, Ethynyl" OR "Ethinyl Estradiol Hemihydrate" OR "Hemihydrate, Ethinyl Estradiol" OR "Ethinyl Estradiol, (8 alpha)-Isomer" OR "Ethinyl Estradiol, (8 alpha,17 alpha)-Isomer" OR "Ethinyl Estradiol, (8 alpha,9 beta,13 alpha,14 beta)-Isomer" OR "Progynon C" OR "Microfollin" OR "Microfollin Forte" OR "Ethinyl-Oestradiol Effik" OR "Ethinyl Oestradiol Effik" OR "Ethinylestradiol Jenapharm" OR "Jenapharm, Ethinylestradiol" OR "Lynoral" OR "Estinyl" OR "Ethinyl Estradiol, (9 beta,17 alpha)-Isomer" |
|  | 8 | "Drospirenone" OR "dihydrospirorenone" OR "6beta,7beta,15beta,16beta-dimethylen-3-oxo-17alpha-pregn-4-en-21,17-carbolacton" OR "1,2-dihydro-spirorenone" OR "1,2-dihydrospirorenone" OR "ZK 30595" OR "ZK30595" |
|  | 9 | "Norethindrone" OR "Norpregneninolone" OR "Norethisterone" OR "Ethinylnortestosterone" OR "Conceplan" OR "Micronor" OR "Norlutin" OR "Nor-QD" OR "Nor QD" OR "NorQD" OR "Norcolut" OR "Norcolute" OR "Norethindrone, (1 beta)-Isomer" OR "Monogest" |
|  | 10 | "Norgestimate" OR "norgestrel oxime acetate" |
|  | 11 | "oestrogen"[All Fields] OR "estrogens"[Pharmacological Action] OR "estrogens"[MeSH Terms] OR "estrogens"[All Fields] OR "estrogen"[All Fields]) AND combination[All Fields] AND ("progesterone"[MeSH Terms] OR "progesterone"[All Fields] |
|  | 12 | "ethinyl estradiol - levonorgestrel" OR "Microgynon" OR "Trikvilar" OR "Triregol" OR "aviane" OR "Gynatrol" |
|  | 13 | #6 OR #7 OR #8 OR #9 OR #10 OR #11 OR #12 |
| Indications OR Medications | 14 | #5 OR #13 |
| Economic analysis | 15 | cost*[ti] |
|  | 16 | benefit cost*[ti] |
|  | 17 | cost savings*[ti] |
|  | 18 | costs*[ti] |
|  | 19 | decision analysis*[ti] |
|  | 20 | economic*[ti] |
|  | 21 | cost minimisation*[ti] |
|  | 22 | cost minimization*[ti] |
|  | 23 | cost utilit*[ti] |
|  | 24 | cost benefit*[ti] |
|  | 25 | cost effectiveness*[ti] |
|  | 26 | cost analy*[ti] |
|  | 27 | economic analy*[ti] |
|  | 28 | economic evaluation*[ti] |
|  | 29 | #15 OR #16 OR #17 OR #18 #19 OR #20 OR #21 OR #22 OR #23 OR #24 OR #25 #26 OR #27 OR #28 |
| Indications OR Medications AND Economic analysis | 30 | #14 AND #29 |
